# Homicide or Happiness: Did Folate Fortification and Public Health Campaigns Influence Homicide Rates and the Great American Crime Decline?

**DOI:** 10.3390/nu16071075

**Published:** 2024-04-06

**Authors:** Stephen J. Schoenthaler, Susan L. Prescott, Alan C. Logan

**Affiliations:** 1 Department of Criminal Justice, College of the Arts, Humanities & Social Sciences, California State University, Turlock, CA 95202, USA; sschoenthaler@csustan.edu; 2 Nova Institute for Health, Baltimore, MD 21231, USA; 3 School of Medicine, University of Western Australia, Perth, WA 6009, Australia; 4 Department of Family and Community Medicine, School of Medicine, University of Maryland, Baltimore, MD 21201, USA

**Keywords:** folate, behavior, criminal justice, nutritional criminology, mental health, microbiome, aggression, biological criminology, ultra-processed foods, violence

## Abstract

The last several years have witnessed a remarkable growth in research directed at nutrition and behavior, with increased interest in the field of nutritional criminology. It is becoming clear that dietary patterns and specific nutrients play an important role in cognition and behavior, including those related to aggression, violence, and antisocial activity. Included in this expanding knowledge base is the recognition that folate, through multiple pathways, including enzymatic reactions and gut microbiome ecology, plays a critical role in central nervous system functioning. These mechanistic advances allow for a retrospective analysis of a topic that remains unexplained—the sudden and unpredicted drop in homicide and other violent crime rates in the United States and other nations in the 1990s. Here, we revisit this marked reduction in homicide rates through the lens of the coincident public health campaign (and subsequent mandatory fortification) to increase folic acid intake. Based on objectively measured blood folate levels through the National Health and Nutrition Examination Surveys, there is little doubt that tissue folate witnessed a dramatic rise at the national level from 1988 through 2000. Drawing from accumulated and emerging research on the neurobehavioral aspects of folate, it is our contention that this relatively sudden and massive increase in tissue folate levels may have contributed to reductions in violent crime in the United States.

## 1. Introduction

Recent years have witnessed a growing interest in the area of nutrition, mental health, and behavior. Most often, these studies have examined dietary factors in relation to depression, anxiety, and psychosis [1,2]. However, investigators have also examined the role of dietary patterns and select nutrients on outcomes such as impulsivity, aggression, violence, and antisocial behavior [3,4,5,6]. Research in the area of attention deficit hyperactivity disorder (ADHD) has helped to shed light on the role of nutrients and dietary patterns in impulsivity [7]. As a result, there is increased scrutiny of the intersection between nutrition and the criminal justice system, in terms of both treatment and prevention [8,9]. For example, omega-3 fatty acids, or a lack thereof, have been linked to impulsivity, suicide by violent means, and national homicide rates [10]. Although folate, part of the B vitamin family, has long-since been understood to play a critical role in supporting normal central nervous system function [11], and folic acid and other related compounds have been reported to improve depression and other mental disorders [12,13], its role in antisocial behavior and aggression is less often discussed.

**Figure 1:**
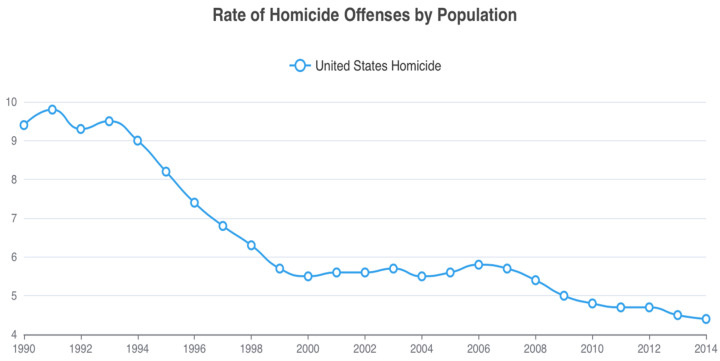
United States homicide rates per 100,000 from 1990 to 2014. From Federal Bureau of Investigation Crime Data Explorer https://cde.ucr.cjis.gov/.

**Figure 2:**
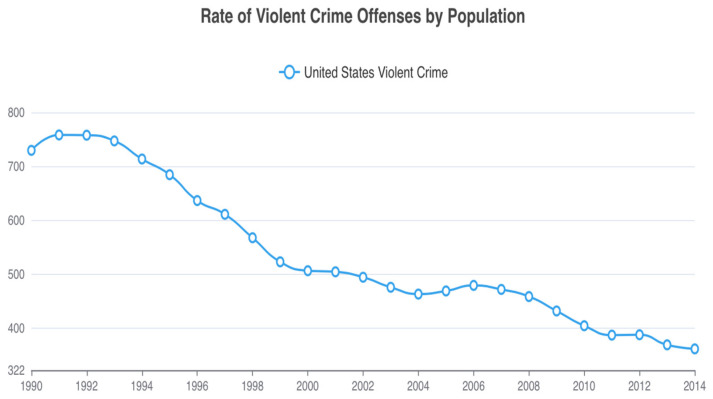
United States violent crime rates per 100,000 from 1990 to 2014. From Federal Bureau of Investigation Crime Data Explorer https://cde.ucr.cjis.gov/.

Here, we suggest that the increased marketing of folic acid supplementation and subsequent food fortification, as a means to reduce neural tube defects, played a part in the relatively sudden and unexpected decrease in homicide rates and other violent crimes in the 1990s. This drop, often referred to as the Great American Crime Decline [14], witnessed a 43% reduction in homicide rates between 1991–2001, and a 34% reduction in violent crime over the same period [15] (Figure 1 and Figure 2). While various social theories have been proposed for the rapid crime decline, no single theory emerges as definitive [16,17]. Also, of relevance to our later discussions on folate and behavior, suicide rates also decreased during this time period, especially among young adults [18] who are historically in the highest risk categories for the commission of homicide and violent crime.

## 2. Folic Acid Recommendations

Beginning in the early to mid-1980s, there was increased recognition that prenatal folic acid supplementation appeared to cut the risk of neural tube defects [19]; in 1989, the results of a large cohort study (*n* = 23,491) showed that folic acid supplementation during the first six weeks of pregnancy resulted in neural tube defects at a rate of 0.9 per 100,000 births, compared to 3.5 per 100,000 for women who did not take a multivitamin or folic acid supplement [20]. The results, published in the Journal of the American Medical Association (JAMA), were highly publicized in front page newspaper articles throughout the United States, with highly regarded experts recommending that all women who are planning a pregnancy take a daily multivitamin inclusive of folic acid [21,22]. In the year following the JAMA article, 1990, Associated Press articles ran in nationwide newspapers, with quotations from experts supporting the use of perinatal multivitamins inclusive of folic acid [23]. This advice filtered into the clinical domain, as exemplified by one notable public health physician who maintained a regular newspaper column: “*If one of my daughters decides to have a child, I hope she will hedge her bets by taking multivitamins containing folic acid even before she becomes pregnant and throughout her pregnancy*” [24].

**Figure 3:**
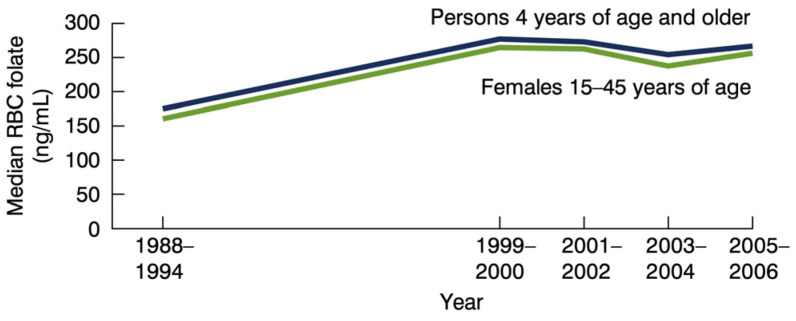
Red blood cell folate trends in the United States. Source: Source: United States Department of Health and Human Services Data Brief #6, May 2008.

**Figure 4:**
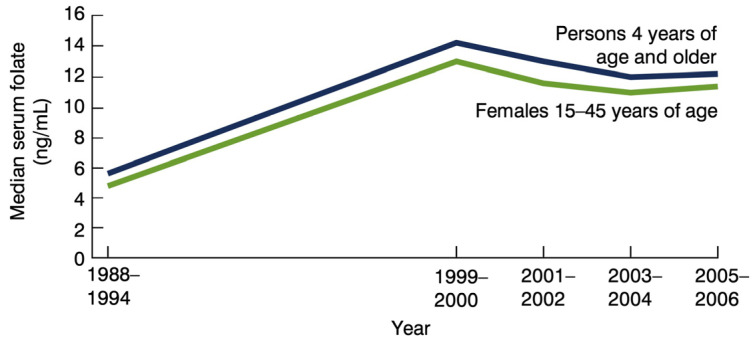
Serum folate trends in the United States. Source: United States Department of Health and Human Services Data Brief #6, May 2008.

The point here is that even before the Centers for Disease Control formally recommended folic acid supplementation (400 mcg) for all women of childbearing age, in 1992 [25], there was already heightened public awareness. It seems safe to assume that although the media-driven awareness of folic acid was directed at women, it appeared to influence folate levels in both genders. We can base this assumption off the National Health and Nutrition Examination Survey (NHANES) evidence showing that the red blood cell folate levels for the entire US population, both men and women, increased significantly from 1988 through the 1990s. By 1999, the total number of persons in the US with what is considered to be “low” red blood cell folate dropped to 2.8%, whereas in 1988, over 30% of the population had low red blood cell folate. However, this average belies disparities, and the fact that almost 60% of Black females (aged 15 to 45) experienced low levels of blood folate by CDC standards; by 2000, the percentage of low folate among Black women had dropped to 12.1%. The median red blood cell folate levels of the entire US population aged 4 and older increased by over 60% from 1988 through 1999 [26] (Figure 3 and Figure 4).

The first attempts at evaluating red blood cell folate levels at the national level, the NHANES II study (1978–1980), revealed that Black males had significantly lower levels of folate than White males [27]. As mentioned, the period between 1988 and 2000 witnessed significant elevations in blood folate among Black adults (although as discussed below, disparities were, and still are, significant) [26]. Notwithstanding the difficulties with comparing disparate national surveys, in 1986 it was reported that 18.4% of all Black male adults consumed dietary supplements [28], while in the period 1988–1994, 26.7% of Black male adults consumed dietary supplements [29], and like most demographic groups, supplement use continued to increase, more recently noted to be 39.3% [30]. Of course, this does not indicate an increased intake of folic acid per se, but does reflect nutritional awareness and a potential increase in large-scale nutrient intake that predates mandatory folate food fortification. Although the mandatory fortification of most flours was effective from 1 January 1998, the Food and Drug Administration (FDA) had already announced mandatory fortification enforcement almost two years earlier, on 29 February 1996 [31].

The idea that nutrients such as folic acid could influence brain and behavior was certainly ongoing when the fields of nutritional neuroscience and nutritional psychiatry emerged in the 1980s, yet were only at the periphery of criminology and the criminal justice system. It is only with advances in research related to nutritional criminology and food crime [8,9] that it is possible to retrospectively examine cases and epidemiological trends that previously escaped explanatory (biological) discourse within the realm of criminal justice [32]. We now turn our attention toward such evidence, including preclinical work, population studies, and mechanistic pathways.

## 3. Folate, Brain, and Behavior

Links between deficiencies of various B vitamins and mental disturbances, including depressive symptoms, anxiety, and irritability, date back to the 1940s [33]. Reports of clinical improvement in mental disorders (including irritability reductions) with B vitamin complex supplements (inclusive of 1500 mcg of folic acid) vs. placebo were first reported in 1954 [34]. In the decades that followed, researchers reported that individuals with mental disorders—ranging from anxiety to schizophrenia, and depression to substance abuse—are more likely to have low blood folate compared to other healthy populations [35,36]. These findings led to the pursuit of possible mechanistic pathways that might explain why folate deficiencies are associated with wide-ranging mental and behavioral symptoms, and why folic acid supplements seem to improve clinical outcomes [35,37]. Folate is at the cornerstone of the one-carbon metabolism. It is involved in numerous enzymatic reactions and metabolic pathways, including mitochondrial function [38] and neurotransmitter production and enzymatic breakdown via S-adenosylmethionine and other methyl reactions. In animal studies, it has been observed that folate deficiencies can lead to alterations in serotonin, dopamine, norepinephrine, and acetylcholine [39,40,41]. Serotonin, for example, appears to promote prosocial behavior by enhancing aversion to harming others [42]. These alterations to serotonergic function and other functional changes in the central nervous system can help explain the loss of impulse control and increased aggression in folate-deficient animals [43,44] (Figure 3 and Figure 4).

Although folic acid has not been studied as a stand-alone intervention in justice-involved individuals, several controlled studies in correctional facilities have examined multivitamin–mineral formulas, inclusive of folic acid. These studies have found reductions in antisocial behavior, rule-breaking, and violence [45,46,47,48]. Combined, this work supports older human studies indicating that the correction of low blood folate with folic acid supplements can improve neurobehavioral outcomes [37].

While folate levels and human violence and aggression have received little epidemiological and clinical attention, such is not the case with suicide. For example, epidemiological research shows that low serum folate is associated with various suicidal behaviors, including previous suicide attempts, suicidal severity, and fatal/non-fatal suicide attempts [49]. In a general population study in the United States, involving prescription claims of over 866,000 adults, a prescription for folic acid was associated with a 44% reduction in suicidal events [50]. These positive results at the general population level were extended to the narrower group of individuals with known psychiatric diagnosis, a history of prior suicidal behavior, and/or receiving a prescription for psychotropic medication; once again, folic acid prescription is robustly associated with lower suicidal event rates in higher suicide risk populations [51]. In addition to calls for controlled trials involving the inclusion of folic acid administration in high-risk psychiatric populations, there is a need to closely examine relationships between folate levels and/or folic acid prescriptions and the means by which completed suicide occurred. Violent methods of suicide (e.g., use of firearms, hanging, jump from high places, immolation, as compared to drug overdose, gas/suffocation, poison) are associated with higher levels of lifetime aggression and impulsivity [52] and distinct neurobiological underpinnings [53]. Included in those biological distinctions related to suicide by violent means are alterations in purinergic signaling and mitochondrial metabolism [54], both of which can be influenced by folate [55].

As mentioned earlier, inadequate omega-3 fatty acid intake (or deficiencies) has been linked to aggression, impulsivity, and suicide risk [56,57]; however, it is worth noting that folate, by way of its influence on methylation reactions, is responsible for the transport of omega-3 fatty acids to target tissue [58]. In animals, the administration of folate can increase plasma and red blood cell levels of omega-3 fatty acids [59], and diets deficient in folate lead to reductions in blood and nervous system tissue omega-3 levels [60,61]. This supports human research involving adults with aggression and hostility, wherein there is a positive association between blood folate levels and higher tissue omega-3 levels [62], and separate research showing that medication-naïve first-episode psychotic patients have a tandem of low folate and omega-3 levels [63]. Thus, in many ways, discussions of omega-3 fatty acids in the context of criminology, including their influence on cell signaling, neuroinflammation, and mitochondrial membrane functioning, are discussions of folate. Taken together, the available research suggests a need for folate (single-nutrient) intervention trials involving vulnerable populations, including justice-involved persons and others at-risk. Only well-designed intervention trials can overcome some of the biases and confounders that have plagued older studies. Recent evidence-based clinical guidelines issued by the World Federation of Societies of Biological Psychiatry and the Canadian Network for Mood and Anxiety Treatments Taskforce have recommended methylfolate for adjunctive use in depression and schizophrenia [64]. Given the potential value, it is surprising that there has been so little research within correctional settings.

## 4. Gut-Brain-Microbiome Links

One exciting area of research that unites discussions of folate and omega-3 fatty acids is the relationship between gut microbes and mental health. Since the development of the first scientific frameworks linking non-pathogenic microbes with mental health and cognition, in the early 2000s [65,66], it has become increasingly clear that the gut microbiome plays an important role in neuropsychiatry [67,68]. There are multiple mechanisms by which gut microbes can influence brain and behavior. These include, but are not limited to, direct communication to the brain via gut-innervating vagus and spinal nerves [69], indirect pathways via influences on systemic immune chemicals (e.g., cytokines) and neuropeptides/hormonal messengers that are well-known to impact mood and behavior [70], influencing the absorption of nutrients [71], and maintaining the integrity of the intestinal barrier (i.e., preventing intestinal permeability or so-called “leaky gut”) [72]. It is worth noting that a compromised intestinal barrier has been linked to increased aggression [73]. Emerging evidence is connecting specific gut microbes and/or microbial signatures with personality features, including temperament [74], propensity for violence [75], and the regulation of emotions [76]. In addition, human intervention studies suggest that targeting the gut microbiome with beneficial microbes might lower aggressive thoughts [77] and impulsivity [78].

Since dietary patterns and nutrients play a critical role in shaping the human microbiome, the status of the gut microbial ecosystem has been postulated to play an important mechanistic role in behavioral outcomes, including those relevant to violence and aggression [3,79]. Strong support for a microbiome-behavior link can be found in fecal transplant studies; that is, when fecal material from animals with nutrient-related or stress-induced dysbiosis (or from human donors with mental disorders) is transplanted into otherwise healthy animals, the recipients have observable neuropsychiatric disturbances, as found in the dysbiotic donors [80,81,82,83]. For example, the transfer of fecal material from human infants with dysbiosis (vs. the transfer of microbiota from healthy animals) to recipient lab animals leads to aggressive-like behavior in recipients lab animals [84]. Mechanistic pathways have been illuminated—recipients of dysbiotic microbiota are noted to display alterations to metabolic pathways, disturbances of the intestinal barrier, changes in neurotransmitter levels (e.g., serotonin and gamma-aminobutyric acid), and micro-RNA alterations in the frontal cortex [85,86,87,88]. While the topic of dietary patterns and nutrition in relation to the gut microbiome is complex, several studies have shown that folate deficiency is associated with disturbances to the gut microbiome in animals [89,90,91], and that folic acid supplementation can prevent dysbiosis in various experimental chronic disease models [92,93]. In sum, there are certainly plausible mechanisms whereby folate deficiency/folic acid supplementation can influence irritability, aggression, impulsivity, and mental outlook.

## 5. Violent Suicide and Homicide—Complexity

It is understood that violent/non-violent suicide and homicide are complex subjects, and rates of both can be influenced by many environmental and socioeconomic factors, [94,95,96,97], regardless of optimal folic acid intakes. The same complexity applies to neural tube defects. It is worth noting that the levels of neural tube birth defects in the United States were already dropping before folic acid food fortification began in 1998, and this is to be expected based on the aforementioned public health campaigns dating back to the 1980s. However, in the two decades following mandatory fortification, rates of (folate-related) birth defects have not declined according to expected trajectories [98]. Despite significant gains in objectively measured serum and red blood cell folate across the United States population from 1988 through 2000, more recent measurements, in the years following fortification, have witnessed a small but meaningful decline (7% for serum and 12% for RBC) in folate levels [99].

The success of nutritional campaigns to lower folate-related birth defects may be dependent upon methyl donors and other nutrients that are involved in folate metabolism. These include nutrients such as vitamins B6 and B12, thiamine, riboflavin, zinc, choline, betaine, and methionine [100]. There are still large segments of the United States population with subpar intake of folic acid [101], including persons living with food insecurity [102,103], and research shows that neighborhood socioeconomic deprivation predicts neural tube defects [104].

It is also important to point out that our discourse is focused on homicide, suicide, and violent crime trends in the United States. In this context, the United States stands as an outlier. For example, the rates of homicide in the United States were, and continue to be, much higher than other high-income countries—in studies based on 2010 and 2015 statistics, homicide in the US was 7 to 8 times higher than in other high-income countries, and rates of violent suicide by firearm were 8 to 10 times higher [105,106]. The extent to which folic acid supplementation influences large-scale outcomes is, of course, dependent upon shifting environmental factors. For example, the less-than-expected declines in post-fortification neural tube defects may be due, at least in part, to rising obesity rates [98]. Overweight and obese individuals have lower serum folate concentrations when compared with individuals with normal weight; this difference may be mediated, at least in part, by the microbiome and changes in intestinal absorption [107]. The intake of energy from low-nutrient, hyper-palatable, and obesogenic ultra-processed foods has increased among adults in the United States in the years following fortification [108,109]. The dominant foods in supermarkets throughout the United States are now ultra-processed—approximately 58% of staples in U.S. leading supermarkets are ultra-processed, which is 41% more than supermarkets in Europe [110]. Moreover, hyper-palatable and ultra-processed foods have been heavily marketed in marginalized communities [111,112] wherein socioeconomic risk factors for violent offending and victimization are already elevated. At the biological level, these foods, and the presence/absence of nutrients, intersect with biopsychosocial vulnerabilities [113]. In the context of nutritional criminology, the increasing dominance of these foods, and their relationship to central nervous system structure and function, may have a detrimental effect on behavior, one that can paste over the potential benefits of folic acid supplementation.

## 6. Conclusions

Multiple theories have been used to explain the sudden and unpredicted drop in homicide and violent crimes through the 1990s. These include, but are not limited to, changes in the illicit drug markets, aggressive policing policies (especially those directed at illegal guns), incapacitation through the expansion of prison populations, and improved economic conditions [15,114]. It is not our contention that these intersecting explanations are without merit. We are simply presenting an additional input. It is our view that an unprecedented chemical experiment—one that produced dramatic shifts in tissue levels of a nutrient chemical at the mass population level—is now supported by biophysiological plausibility as a determinant of behavior. Of course, our observations of coincident increases in tissue folate and decreases in violent crime, throughout the 1990s, are merely associations and not proof of causation. However, the idea that a relatively sudden and significant rise in a blood chemical is without any neurobehavioral consequence (beyond those associated with neural tube defects) seems to be the more implausible consideration. At this stage, controlled intervention trials using folic acid, and other nutrients involved in folate metabolism, seem overdue [8,9].

## Data Availability

No new data were created or analyzed in this study. Data sharing is not applicable to this article.
